# The cost-effectiveness of treatments for attention deficit-hyperactivity disorder and autism spectrum disorder in children and adolescents: a systematic review

**DOI:** 10.1007/s00787-021-01748-z

**Published:** 2021-03-09

**Authors:** Filipa Sampaio, Inna Feldman, Tara A. Lavelle, Norbert Skokauskas

**Affiliations:** 1 Department of Public Health and Caring Sciences, Uppsala University, Husargatan 3, P.O Box 564, 751 22 Uppsala, Sweden; 2 Department of Epidemiology and Global Health, Umeå University, Umeå, Sweden; 3 Center for the Evaluation of Value and Risk, Institute for Clinical Research and Health Policy Studies, Tufts Medical Center, Boston, MA USA; 4 Regional Centre for Child and Youth Mental Health and Child Welfare, IPH, Faculty of Medicine and Health Sciences, Norwegian University of Science and Technology, Trondheim, Norway; 5 Child and Adolescent Mental Health Services, St. Olav Hospital, Trondheim, Norway

**Keywords:** Neurodevelopmental disorders, Attention deficit-hyperactivity disorder, Autism spectrum disorder, Cost-effectiveness analysis, Treatment, Intervention

## Abstract

Economic evaluations can help decision makers identify what services for children with neurodevelopmental disorders provide best value-for-money. The aim of this paper is to review the best available economic evidence to support decision making for attention deficit-hyperactivity disorder (ADHD) and autism spectrum disorder (ASD) in children and adolescents. We conducted a systematic review of economic evaluations of ADHD and ASD interventions including studies published 2010–2020, identified through Econlit, Medline, PsychINFO, and ERIC databases. Only full economic evaluations comparing two or more options, considering both costs and consequences were included. The quality of the studies was assessed using the Drummond checklist. We identified ten studies of moderate-to-good quality on the cost-effectiveness of treatments for ADHD and two studies of good quality of interventions for ASD. The majority of ADHD studies evaluated pharmacotherapy (*n* = 8), and two investigated the economic value of psychosocial/behavioral interventions. Both economic evaluations for ASD investigated early and communication interventions. Included studies support the cost-effectiveness of behavioral parenting interventions for younger children with ADHD. Among pharmacotherapies for ADHD, different combinations of stimulant/non-stimulant medications for children were cost-effective at willingness-to-pay thresholds reported in the original papers. Early intervention for children with suspected ASD was cost-effective, but communication-focused therapy for preschool children with ASD was not. Prioritizing more studies in this area would allow decision makers to promote cost-effective and clinically effective interventions for this target group.

## Introduction

Neurodevelopmental disorders (NDDs) are multifaceted conditions characterized by impairments in cognition, communication, behavior and/or motor skills [1, 2]. Autism spectrum disorder (ASD) and attention deficit-hyperactivity disorder (ADHD) are the two most common NDDs in childhood, with ADHD and ASD affecting 7% and 1–2% of children worldwide, respectively [3]. ADHD typically begins before adolescence and is characterized by inattention and disorganization, with or without hyperactivity–impulsivity, and causing impaired functioning in school, home and social settings [1]. ASD typically appears before the age of 3 years and is characterized by impairment in social interactions and communication skills, as well as the presence of restricted and stereotypical behaviors [1].

The societal burden of childhood NDDs is substantial: the average annual cost of ADHD per child in Europe was between $7,369 (€5,733) and $18,616 (€14,483) (2012 prices) with direct costs accounting for 60% of the total. The largest direct costs were from psychological support (46%) and pharmacotherapy (26%). Among indirect costs, 65% were due to caregiver lost productivity [4–6]. Adult ADHD is also associated with costly negative outcomes, including criminality, employment, problems in social skills, and comorbid psychiatric disorders [7].

ASD is also associated with a large economic burden. Children aged 3–17 years with ASD have $3,020 (€2,168) higher annual healthcare costs and $14,061 (€10,096) higher non-healthcare costs, compared to children without ASD, including $8,610 (€6,182) higher annual school costs (2011 prices) [8]. Costs associated with ASD persist into adulthood due to the substantial costs resulting from adult care (home, community and residential) and lost productivity for both individuals with ASD and their parents; with the lifetime per capita incremental societal cost of ASD estimated as $3.2 (€2.9) million (2003 prices) [9]. Given the large financial burden of both disorders for individuals, families and society, both in the shorter and longer term, it becomes crucial to include and clearly discriminate the full spectrum of costs associated with these disorders.

Early identification of NDDs is critical to the wellbeing of children, their families, and society. For instance, children with ASD who receive appropriate and timely interventions need fewer additional supportive services, including applied behavioral analysis, occupational, physical, and speech therapy, during childhood [10], and these benefits may persist into improved functioning as an adult [11]. Evidence from economic evaluations can help decision-makers identify which services are a good investment, contributing to the health of the child, and providing a sound use of limited societal resources [12].

The Lancet Psychiatry Commission [13] emphasizes the need to not only focus on the effectiveness of mental health services, but also on their economic benefits. Despite this, there are few reviews of economic evaluations of ADHD and ASD interventions [14–16]. Beecham et al. [16] emphasized that little is known about the economic implications of ASD treatments. A review by Wu et al. [17] on the cost-effectiveness of pharmacotherapies for ADHD concluded that these were cost-effective compared with no treatment or behavioral therapy. However, the use of medication for ADHD entails disadvantages, including adverse effects, a heightened chance of relapse by discontinuation, and unknown effects in the long-term [18]. Psychosocial and behavioral interventions, including classroom, family and child focused interventions, are also recommended treatments for ADHD [19], and have demonstrated to be effective in improving child behavior and functional outcomes [20, 21]. These can be implemented alone or in conjunction with pharmacological therapies. Psychosocial and behavioral interventions have also demonstrated to be beneficial to children and adolescents in terms of intellectual functioning, behavior, language development, acquisition of daily living skills and social functioning [22, 23]. Yet, no reviews of economic evidence include these options.

The aim of this review is to provide a comprehensive overview of the most recent literature on the health economic evidence for ADHD and ASD interventions for children and adolescents. In addition, we have appraised the quality of the studies included, discussed methodological challenges and ways to mitigate them. By summarizing the best available evidence for this group of children, we aim to support policy makers and other interested stakeholders in identifying solutions to improve the wellbeing of these children, as well as identifying areas for improvement in future studies.

## Methods

### Search strategy and selection criteria

This review adhered to the guidelines in the preferred reporting items for systematic reviews and meta-analyses (PRISMA) statement for reporting systematic reviews [24] and proposed methods for reporting economic evidence in systematic reviews [25] (Prospero registration number CRD42020192409). We performed an English language search on Econlit, Medline, PsychINFO, and ERIC databases for papers published 2010–2020. This period was chosen to ensure that the studies were relevant to changes in diagnostic criteria for ASD and ADHD encompassed in the Diagnostic and Statistical Manual of Mental Disorders, Fifth Edition (DSM-5) released in 2013, as well as the most recent treatment strategies and clinical practice guidelines [19, 26]. The following search terms were used: “economic evaluati*” OR “cost benefit” OR “cost effectiv*” OR “cost utility” OR “cost–benefit” OR “cost-effectiv*” OR “cost-utility” OR “cost-minimi*” OR “cost minimi*” AND "neurodevelopmental disorder*" OR “pervasive developmental disorder” OR "ADHD" OR "attention deficit hyperactivity disorder" OR “attention deficit disorder” OR “ADD” OR autism OR “ASD” AND child* OR adolescen* OR teen*. An additional search was conducted in the Pediatric Economic Database Evaluation (PEDE) Registry [27].

Inclusion criteria comprised: (1) full economic evaluations comparing two or more options, including both costs and consequences; (2) studies evaluating treatment strategies (either pharmacological or psychosocial/behavioral strategies) targeting ADHD or ASD; (3) studies evaluating interventions targeting either the child alone or both child and parent(s). The review excluded systematic reviews, editorials and conference abstracts, and studies only targeting comorbidities or other problems in children and adolescents with ADHD/ASD.

Two reviewers (FS, IF) independently screened titles and abstracts to assess relevance based on inclusion and exclusion criteria. To ensure consistency in authors’ assessments, 20% of all articles reviewed by each reviewer were randomly selected to be reviewed by the other reviewer. The author agreement on article inclusion was estimated based on inter-rater reliability, producing a Cohen’s kappa of 0.83, reflecting good agreement [28]. Abstracts included were next assessed for full-text inclusion. Full-text articles fulfilling inclusion criteria were selected for data extraction.

### Data extraction

The following data were extracted using a tailored sheet and summarized in a narrative format: author/year, country, setting, population, study type, intervention, comparator, follow-up/time horizon, type of evaluation, perspective of the economic analysis and types of costs included, outcomes, instruments used, and summary of results. The extraction sheet was piloted for completeness using three sample studies. Two authors extracted data (FS, IF), and 20% of the articles included were randomly selected for revision by another author (TL). Discrepancies in study selection and data extraction were resolved through discussions with all the authors.

### Quality assessment

We assessed the quality of studies using the 10-item Drummond checklist [12]. Two authors completed the checklist for all studies (FS, IF), and a random 20% was reviewed by a third author (TL). Discrepancies were resolved in discussions with all the authors. We created a scoring system, and calculated an average score across the 10 items, with each item weighted equally [29]. All items have three potential responses “yes”, “unclear” and “no”, which were scored 1, 0.5 and 0, respectively. Items 6 and 7 have the additional potential response “not applicable”. When this occurred, these items were excluded from the calculation. Studies were classified into good (score 0.8–1.0), moderate (score 0.6–0.79) and poor quality (score < 0.59).

### Economic evaluation frameworks

We classified studies according to the type of economic evaluation performed. The most common types of evaluations are cost–benefit analyses (CBA), cost-effectiveness analyses (CEA), and cost-utility analyses (CUA). All types follow similar principles and value costs in monetary terms, differing mainly on the measurement of health outcomes. In CBA, both costs and outcomes are measured in monetary units. In CEA, outcomes are measured in clinically meaningful units, such as proportion of people responding to ADHD treatment. In CUA, outcomes are measured as Quality-Adjusted Life-Years (QALYs), which combine both mortality and morbidity impacts. QALYs are calculated by multiplying the length of time spent in a particular health state by a “utility weight”, which designates the “preference” society has for that health state. Weights usually range between 0, denoting death, and 1, denoting full health. CUA allows value-for-money judgments to be made, and allows the comparison of the cost-effectiveness of interventions across different disorders. Cost-minimization analysis (CMA), which compares costs between two interventions, is less common, and is only employed when two interventions have equal outcomes. The evaluation then reduces to a cost-analysis, whereby the cheaper intervention is preferred. We included CBAs, CEAs, CUAs and CMAs in our review.

We also identified the method for measuring health state utilities needed for the estimation of QALYs. Utilities can be calculated using direct valuation methods, such as the Time Trade-Off (TTO), and indirect methods. The TTO is a choice-based method that establishes for an individual how much time in full health is equivalent to a specified period of time spent in a particular ill-health state. Indirect methods facilitate indirect elicitation of utilities and estimation of QALYs with inbuilt algorithms that allow for the derivation of utility weights based on participant responses. The indirect approach involves the use of multi attribute utility instruments (MAUI). A commonly used instrument is the Euroqol-5 dimensions (EQ5D). Cost-effectiveness guidelines in most countries recommend indirect methods, and the use of a generic health-related quality-of-life (HRQoL) instrument to measure QALYs [30, 31].

Each study was also classified as to whether it was conducted through primary data collection, or simulation modeling. Economic evaluations are classified as within-trial studies when the evaluation piggy-backs onto a trial, usually a randomized controlled trial (RCT). Alternatively, economic simulation modeling studies are widely used to synthesize data from multiple sources. Models can be used to incorporate all sources of evidence, and to estimate the long-term impacts of interventions, which often cannot be captured in time-limited trials. Models are the main form of evaluation used by international decision-making agencies.

## Results

### Search results

**Fig. 1:**
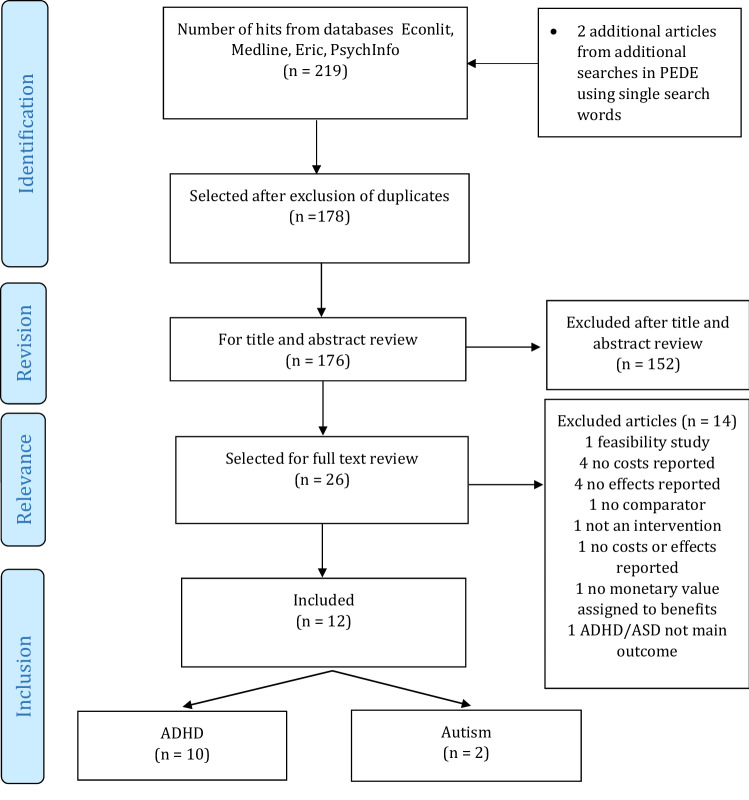
PRISMA flow diagram of study selection process

The search strategy produced 176 unique publications, and 2 additional papers were found via PEDE. After screening all titles and abstracts, 26 articles advanced to full-text review. Of these, we excluded 14 studies that were not full economic evaluations and reported only costs (*n* = 4) or only outcomes (*n* = 4), did not report costs or outcomes (*n* = 1), did not include a comparator (*n* = 1), were not an evaluation (*n* = 2), had no monetary value assigned to benefits (*n* = 1), and did not target ADHD/ASD (*n* = 1). Twelve studies fulfilled the inclusion criteria and were selected for data extraction. Figure 1 shows the PRISMA diagram of the study selection process.

### Quality assessment

Most studies targeting ADHD were of good quality (*n* = 8) [32–39], and two studies were of moderate quality [40, 41]. Both studies targeting ASD were of good quality [42, 43]. The most common reason for not receiving full points was due to the lack of inclusion of uncertainty around estimates of costs and consequences [32, 35, 40, 41] (see Table 1).

**Table 1:** Quality assessment of the studies included

Criteria author	1. Was a well-defined question posed in answerable form?	2. Was a comprehensive description of the competing alternatives given?	3. Was the effectiveness of the programs or services established?	4. Were all the important and relevant costs and consequences for each alternative identified?	5. Were costs and consequences measured accurately in appropriate physical units prior to valuation?	6. Were the cost and consequences valued credibly?	7. Were costs and consequences adjusted for differential timing?	8. Was an incremental analysis of costs and consequences of alternatives performed?	9. Was uncertainty in the estimates of costs and consequences adequately characterized?	10. Did the presentation and discussion of study results include all issues of concern to users?	Score
ADHD											
Tran et al. [40]	✓	✓	✓	x	✓	✓	x	✓	x	✓	0.70
Sonuga-Barke et al. [32]	✓	✓	✓	✓	Unclear	Unclear	n.a.	✓	Unclear	✓	0.83
Zimovetz et al. [33]	✓	✓	✓	✓	✓	✓	✓	✓	✓	✓	1.00
Sohn et al. [34]	✓	✓	✓	✓	✓	✓	n.a.	✓	✓	✓	1.00
Maia et al. [35]	✓	✓	✓	✓	✓	✓	✓	✓	x	✓	0.90
Lachaine, et al. [36]	✓	✓	✓	✓	✓	✓	n.a.	✓	✓	✓	1.00
Schawo et al. [37]	✓	✓	x	✓	✓	✓	✓	✓	✓	✓	0.90
van der Schans, et al. [38]	✓	✓	Unclear	✓	✓	✓	✓	✓	✓	✓	0.95
Erder et al. [41]	✓	✓	✓	✓	✓	✓	n.a.	✓	x	x	0.78
Sikirica et al. [39]	✓	✓	✓	✓	✓	✓	n.a.	✓	✓	✓	1.00
Autism spectrum disorder											
Byford et al. [42]	✓	✓	✓	✓	✓	✓	n.a.	✓	✓	✓	1.00
Penner et al. [43]	✓	✓	x	✓	✓	✓	✓	✓	✓	✓	0.90
% Meet criterion	100%	100%	75%	83%	92%	92%	83%	100%	67%	92%	

### Overview of the studies

Ten of the studies evaluated treatments in different subpopulations of children and/or adolescents with ADHD, and two studies evaluated strategies for preschool aged children and toddlers with ASD. Four of the 12 studies came from the USA, 3 from the UK, 2 from the Netherlands, and 1 each from Canada, Sweden, and Brazil. The main characteristics of the studies are summarized in Table 2, and methods and results are summarized in Table 3. All costs were converted to 2020 US$.

**Table 2:** Characteristics of the studies included

Author and year	Country	Population	Setting	Study type	Intervention	Comparator	Follow-up/time horizon
ADHD							
Tran et al. [40]	USA	7–11 y.o. children with ADHD-I	Schools	RCT	Psychosocial program Child Life and Attention Skills (CLAS): included integrated parent, teacher, and child components (90-min parent group meetings, 30-min individual meetings with the parents and child, 90-min child group meetings, 30-min teacher consultation meetings)	1. Psychosocial program parent-focused treatment (PFT) as active treatment control arm, only incorporated the parent component from the CLAS program (90-min parent group meetings, 30-min individual meetings with the parents and child); 2. TAU: conventional treatment by community providers that was available to all participants	13 weeks (3 months)
Sonuga-Barke et al. [32]	UK	Preschool children with ADHD	Home and outpatient clinic settings	RCT	1. New Forest Parenting Program (NFPP): 12-week, 1.5 h sessions; 2. Incredible Years (IY) parenting program: 12-week 2–2.5 h sessions and weekly phone calls	TAU (standard patterns of preschool ADHD care available ranging from parent training and education to very little support)	6 months
Zimovetz et al. [33]	UK	Children and adolescents with ADHD with inadequate response to methylphenidate	Outpatient health	Modeling	Lisdexamfetamine dimesylate (LDX)	Atomoxetine (ATX)	1 year
Sohn et al. [34]	USA	Children and adolescents with ADHD who failed initial stimulant treatment	Outpatient health	Modeling	Atypical antipsychotics (AAPs): aripiprazole, olanzapine, paliperidone, quetiapine, risperidone, ziprasidone	1. Clonidine/guanfacine; 2. Atomoxetine	1 year
Maia et al. [35]	Brazil	6–17 y.o. with ADHD	Outpatient health	Modeling	1. Immediate-release methylphenidate (IR-MPH) (Children); 2. IR-MPH (Adolescents)	Natural course of disease (do-nothing)	6 years
Lachaine, et al. [36]	Canada	6–12 y.o. with ADHD with suboptimal response to stimulants	Outpatient health	Modeling	Non-stimulant GXR-ER (guanfacine extended-release) as adjunctive therapy to a long-acting stimulant	Long-acting stimulant monotherapy	1 year
Schawo et al. [37]	Netherlands	6 y.o. with ADHD with suboptimal response to IR-MPH	Outpatient health	Modeling	Methylphenidate osmotic-release oral system (MPH-OROS)	IR-MPH	12 years
van der Schans, et al. [38]	Netherlands	8 y.o. with suboptimal response to IR-MPH	Outpatient health	Modeling	1. IR-MPH	ER-MPH options included: 1. MPH-OROS; 2. Equasym XL/Medikinet CR	10 years
Erder et al. [41]	USA	6–18 y.o. with ADHD	NR	Modeling	Non-stimulant GXR-ER	Atomoxetine	1 year
Sikirica et al. [39]	USA	6–17 y.o. with ADHD with suboptimal response to stimulant monotherapy	Outpatient health	Modeling	Non-stimulant GXR + stimulant	Stimulant monotherapy	1 year
Autism spectrum disorders							
Byford et al. [42]	UK	Preschool children with autism	Community-preschools	RCT	Pre-School Autism Communication Trial (PACT) communication-focused intervention + TAU: includes assessment session followed by fortnightly one-to-one clinic sessions for six months. 2.5 h sessions conducted between therapist and parent with the child present, followed by monthly booster sessions for six months (max. 19 sessions). 30 min daily home practice between sessions	TAU: provided by local services, commonly including pediatricians and speech and language therapists, alongside a variety of other health, social care and education-based services	13 months
Penner et al. [43]	Canada	Toddlers aged 15–36 months with undifferentiated developmental concerns	Outpatient health	Modeling	Early Start Denver Model Intensive (ESDM-I): children receive the intervention delivered by a trained therapist for 20 h per week over a 2-year period, at pre-diagnosis	1. Early Start Denver Model Parent-delivered (ESDM-PD): children receive 1 h per week of therapist intervention over 12 weeks, with the remainder of intervention delivered by parents in the home environment; 2. Status Quo (current practice): children receive the Autism Intervention Program after diagnosis, which provides an Early Intensive Behavioural Intervention (EIBI) consisting of at least 20 h of therapy per week for at least 6 months, and often upwards of 2 years. The EIBI is provided to 37% of children with ASD at the ‘‘more severe end of the spectrum"	Until 65 y.o.

*AAP atypical antipsychotics, ADHD *attention deficit/hyperactivity disorder, *ASD *autism spectrum disorders, *ATX *atomoxetine, *CLAS* Child Life and Attention Skills, *EIBI*  Early Intensive Behavioural Intervention, *GP *General Practitioner, *GXR *guanfacine extended-release, *IR-MPH *immediate-release methylphenidate, *LDX *lisdexamfetamine dimesylate, *MPH*-*OROS methylphenidate *osmotic-release oral system, *NFPP* New Forest Parenting Program, PACT Pre-School Autism Communication Trial, PFT parent-focused treatment, *RCT *randomized controlled trial, *TAU *treatment-as-usual, *XR-MPH *extended-release methylphenidate

**Table 3:** Summary of methods and results of the studies included

Author, year	Type of evaluation	Analysis perspective	Health outcomes reported (instruments)	Costs included	Results (US$, 2020)^a^	Quality
ADHD						
Tran et al. [40]	CEA	Modified societal	ADHD-I cases resolved based on parent and teacher completed CSI	Intervention costs (clinician time to run interventions, coordination time and supplies, teacher time); TAU: other healthcare costs (medication, psychotherapy from community providers), childcare, parents' time attending meetings and helping children with homework	Both PFT and CLAS had significantly less ADHD-I cases resolved than TAU. PFT had the lowest cost per patient (ICERs per ADHD-I cases resolved: $4,672 for CLAS versus TAU, $3,772 for PFT versus TAU, and $5,838 for CLAS versus PFT).	Moderate
Sonuga-Barke et al. [32]	CMA	Societal	SNAP-IV mean scores	Health care services (health clinics, health visitors, GPs, pediatric and mental health services); extra educational provision (school nurses, educational psychologist); social services and parental time off work	No differences between NFPP, IY and TAU with regards clinical effectiveness. Individually delivered NFPP was less costly to deliver than IY. No costs estimated for TAU	Good
Zimovetz et al. [33]	CUA	NHS	QALY (EQ5D)	Health care costs (GP, psychiatrist, pediatrician, nurse, blood work and other exams) and drug costs	From the perspective of the UK NHS, LDX provides a cost-effective treatment option for children and adolescents who are inadequate responders to methylphenidate (ICER = $3,017/QALY at a WTP of $33,485/QALY)	Good
Sohn et al. [34]	CUA	Third party payer	QALY (PedsQL)	Health care costs (hospitalizations, emergency room visits, outpatient visits), prescription drug costs	AAPs were less effective and more costly than clonidine/guanfacine and atomoxetine	Good
Maia et al. [35]	CUA	Public health system	QALY (HUI)	Treatment costs (drug and outpatient)	IR-MPH treatment of children and adolescents is cost-effective for ADHD patients from the Brazilian public health system perspective. ICER = $10,070/QALY (children); $13,145/QALY (adolescents) at a WTP of $38,264/QALY	Good
Lachaine, et al. [36]	CUA	Societal	QALY (TTO and VAS)	Health care services (primary care, mental health visits, pharmacy fills, emergency department visits and hospitalizations), medication, productivity losses for parents	GXR as an adjunctive therapy to long-acting stimulants is a cost-effective strategy compared to long-acting stimulant monotherapy in the treatment of children with ADHD. 100% cost-effective at WTP = $ 45,677/QALY. ICER = $21,669/QALY	Good
Schawo et al. [37]	CUA	Societal	QALY (EQ5D)	Treatment costs (drug and consultations), outpatient costs, institutionalization, psychoeducation, parent/teacher and home training, behavior therapy child, social skills training, physical therapy, criminal justice, educational support, special education, productivity losses parents, out-of pocket medical expenses	For children responding suboptimally to treatment with IR-MPH, the beneficial effect of MPH-OROS on compliance is worth the money. The probability of OROS being cost-effective ranges between 93 and 99%. Cost savings of $7,938	Good
van der Schans, et al. [38]	CUA	Societal	QALY (TTO and VAS)	Medication costs, other direct costs (medical consultation, behavioral intervention, and special education) and indirect costs (due to direct medical costs of the mother, absenteeism and presenteeism)	Switching suboptimally treated patients from IR-MPH to MPH-OROS or Equasym XL/Medikinet CR led to per-patient cost-savings of $5,748 and $7,390, respectively, over a 10-year treatment span	Good
Erder et al. [41]	CEA, CUA	Third party payer	Proportion of responders, defined as patients with 25% reduction in ADHD-RS-IV total score, QALY (EQ5D)	Drug costs and other medical costs	GXR is cost-effective compared with ATX for the treatment of ADHD in children and adolescents. ICER: $12,357/QALY gained (WTP of $50,000/QALY); $1,005 per responder	Moderate
Sikirica et al. [39]	CUA	Third party payer	QALY (TTO and VAS)	Drug costs and other medical costs	The adjunctive therapy of GXR with stimulants is a cost-effective treatment compared to stimulant monotherapy. ICER = $37,780/QALY. 94.6% probability of cost-effectiveness at a WTP of $50,000/ QALY	Good
Autism spectrum disorders						
Byford et al. [42]	CEA	Societal	Clinically meaningful improvement in ADOS-G social communication algorithm score	Health care (intervention, speech and language, community health, meds, hospital-based services), education, childcare, social services, parental productivity losses, parental out-of-pocket expenses, informal care	Non-significant improvements in outcome. Larger health, education and social services for PACT + TAU versus TAU. Total cost lower when burden on parents is included. No evidence for investment in PACT + TAU	Good
Penner et al. [43]	CEA	Societal	Dependency-free life years (DFLYs)^b^	Intervention costs, special education, special services at home, income support and healthcare, caregiver costs, productivity losses for children	Pre-diagnosis ASD-targeted intervention may be associated with cost savings (between $21,011 and $52,985) compared to current Ontario service models	Good

*AAP atypical antipsychotics, ADHD attention deficit/hyperactivity disorder, ADHD-RS-IV *ADHD rating scale, *ADOS-G *autism diagnostic observation schedule-generic, ASD Autism spectrum disorder, *ATX* atomoxetine, *CEA *cost-effectiveness analysis, *CLAS* Child Life and Attention Skills, *CMA *cost-minimization analysis, *CSI *Child Symptom Inventory, *CUA *cost-utility analysis, *EQ5D *Euroqol 5 dimensions, *GXR* guanfacine extended release, *HUI *health utilities index, *ICER* incremental cost-effectiveness ratio, *IR-MPH* immediate-release methylphenidate, *LDX* Lisdexamfetamine dimesylate, *MPH-OROS* methylphenidate osmotic-release oral system, *NFPP* New Forest Parenting Program, *NHS* National Health Service, *PACT* Pre-school Autism Communication Trial, *PedsQL* Pediatric Quality of Life Inventory, *PFT* parent-focused treatment, QALY quality adjusted life year, *SNAP-IV *Swanson Nolan and Pelham, *TAU* treatment as usual, *TTO *time trade off, *VAS *visual analogue scale, *WTP *willingness-to-pay

^a^All costs converted to 2020 US$ from original currency using a conversion rate based on Purchasing Power Parities (PPP) for gross domestic product from http://eppi.ioe.ac.uk/costconversion/default.aspx

^b^A DFLY was defined as a year of life with a similar level of independence as a typically developing individual

If more than one perspective, the broadest perspective was indicated

### Attention deficit-hyperactivity disorder

#### Interventions and comparators

Among the high-quality studies, we identified one study that evaluated behavioral parenting interventions and seven studies that evaluated pharmacotherapy. The former compared the New Forest Parenting Program (NFPP) and the Incredible Years (IY) to TAU, defined as different levels of standard support, parent training and education [32]. Three pharmacotherapy studies evaluated the economic value of different formulations of methylphenidate (MPH), a stimulant medication [35, 37, 38]. Two studies compared MPH with an immediate-release (IR) preparation to different formulations of extended-release (ER) MPH [37, 38], and one study compared it to the natural course of disease [35]. Four studies evaluated the economic value of non-stimulant medications: guanfacine extended-release (GXR-ER), and lisdexamfetamine dimesylate (LDX) [33, 34, 36, 39]. Two investigated the added value of non-stimulant therapy (GXR-ER) adjunctive to stimulant therapy compared to stimulant monotherapy [36, 39]. One study compared non-stimulant medications (LDX) to atomoxetine (ATX) [33]; and one study compared AAPs (aripiprazole, olanzapine, paliperidone, quetiapine, risperidone, and ziprasidone) with other non-stimulant medications (ATX and clonidine/guanfacine) [34].

Among the moderate-quality studies, one study evaluated a psychosocial program including parent, teacher and child components and compared it to the same program with a parent-only component and to treatment-as-usual (TAU), which consisted of conventional treatment by community providers [40]; and one study compared non-stimulant medication (GXR-ER) to atomoxetine (ATX) [43].

#### Evaluation framework and measures of effectiveness

Among the good-quality studies, seven were modeling exercises [33–39] and simulated the costs and benefits of pharmacotherapies over different time horizons. Four of these studies had a time horizon of 1 year [33, 34, 36, 39]**,** and three had time horizons between 6 and 12 years [35, 37, 38]**.** One study was an RCT with a time horizon of 6 months [32]**.**

Most studies (*n* = 7) were CUAs and used QALYs as their primary outcome. QALYs were calculated using direct (*n* = 3) and indirect methods (*n* = 3). Among those using indirect methods, two studies used the EQ5D generic HRQoL instrument (parent proxy) [33, 37], and one study used the Health Utilities Index (HUI) (parent proxy) [35]. Three studies included utilities derived using direct methods from the general public (TTO or a visual analogue scale (VAS)) [36, 38, 39]. One study estimated QALYs based on utilities sourced from the literature [34]. One study [32] conducted a CMA, and used mean scores on a validated measure of ADHD symptoms, the SNAP-IV (Swanson, Nolan, and Pelham Questionnaire) [44], as the outcome.

Both moderate-quality studies [41,41] were CEA and used the proportion of treatment responders as the outcome, with response defined as improvement in scores from a rating scale based on DSM-IV criteria. One of these studies also included QALYs measured using the EQ5D [41]. Both studies had short time horizons up to 1 year.

#### Costing perspectives

Among the good-quality studies, four out of the ten studies reported taking a societal perspective [32, 36–38], and included both healthcare costs and some form of caregiver productivity losses, mostly due to absence from work. Three of these [32, 37, 38] also included education. Five studies reported a payer perspective [34, 39], a public health system [35] and a UK national health service (NHS) perspective [33] and included only drug and other healthcare costs.

Of the moderate-quality studies, one [41] reported a payer perspective and included drug and other healthcare costs; and one [40] reported taking a US modified societal perspective and included both healthcare costs and parents’ time costs for attending meetings and providing homework help.

#### Results of the studies

Among the good-quality studies targeting ADHD, one found no differences in outcomes between two group-based parenting interventions, NFPP and IY, and TAU [32], with NFPP being cheaper to deliver than the IY. Among the studies evaluating pharmacotherapy, Maia et al. [35] found that treatment with IR-MPH was cost-effective for children and adolescents with ADHD compared to the natural course of disease (do-nothing) (ICER = $10,070/QALY for children and $13,145/QALY for adolescents). Two studies [37, 38] found that treatment with ER-MPH for children responding suboptimally to IR-MPH improved quality-of-life and saved money compared to no treatment. Two studies evaluating non-stimulant therapy adjunctive to stimulant therapy demonstrated its cost-effectiveness for treating children with suboptimal response to stimulant monotherapy (ICERs ranging between $21,669/QALY [36] and $37,780/QALY [39]). A study comparing two non-stimulant drugs [33] demonstrated that non-stimulant LDX (ICER = $3,017/QALY) was cost-effective, compared to non-stimulant ATX for those with inadequate response to MPH. Sohn et al. [34] concluded that APPs were less effective and more costly than other non-stimulant drugs such as clonidine/guanfacine and ATX for children and adolescents with ADHD who failed initial stimulant treatment.

Among the moderate-quality studies, Tran et al. [40] found that both a parent-focused treatment and an integrated parent, teacher and child treatment for 7- to 11-year-olds with inattentive type ADHD cost more but resolved more ADHD cases than community-based TAU, with the parent-focused treatment being the cheapest alternative. Erder et al. [41] demonstrated that non-stimulant GXR-ER (ICER = $12,357/QALY) was cost-effective compared to non-stimulant ATX for those with inadequate response to MPH.

#### Autism spectrum disorder

Both studies evaluating ASD interventions were of good quality. Byford et al. [42] investigated the within-trial cost-effectiveness of adding a communication-focused intervention for preschool children and their parents to TAU, compared to TAU alone. TAU consisted of standard-provided local services including pediatricians and speech and language therapists, alongside other health, social care and education-based services. Costs were collected from a societal perspective over 13 months and included healthcare, education, childcare, and social services costs, as well as parental out-of-pocket expenses, productivity losses and informal care costs. The study showed a non-significant improvement in autism symptoms (measured by the Autism Diagnostic Observation Schedule-Generic (ADOS-G) social communication score [45]), and significantly higher health, education and social service use costs for the intervention plus TAU compared with TAU alone. The difference in total costs became smaller and non-significant when adding parental indirect costs, however, results did not provide support for investing in the intervention.

Penner et al. [43] modeled the cost-effectiveness of two pre-diagnosis management strategies for toddlers with early warning flags of ASD. The study compared two generic developmental early intervention (EI) programs to the current practice in Ontario*,* which involved service models offering limited access to EI after diagnosis and to a small fraction of children with ASD at the more severe end of spectrum. These two management strategies combined behavioral and developmental approaches into treatment, with one being delivered fully by a therapist (Early Start Denver Model Intensive (ESDM-I)) and the other being delivered by both therapists and parents (Early Start Denver Model Parent-delivered (ESDM-PD)) at pre-diagnosis. The perspective was societal, and included costs for the intervention, special education, special services at home, and healthcare, as well as children’s lost productivity during adulthood, and costs of caregiver time to support the child. Costs and benefits were modeled through age 65. The study reported that EI targeting children with suspected ASD may be associated with cost-savings compared to current practice in Ontario, Canada.

## Discussion

This review aimed to synthetize the economic evidence for ADHD and ASD interventions in children and adolescents. In the last decade, 12 studies of good to moderate quality on the cost-effectiveness of ADHD and ASD interventions were published. Among the studies of good quality targeting ADHD, seven evaluated the economic value of pharmacotherapy and one investigated behavioral parenting interventions. Two economic evaluations of ASD interventions of good quality were published: a communication intervention and EI. Among the studies of moderate quality targeting ADHD, one evaluated pharmacotherapy and one investigated a psychosocial intervention.

Overall, available good to moderate-quality studies support the cost-effectiveness of behavioral interventions for younger children with ADHD. Studies also demonstrated positive clinical and economic results for stimulant medication (LDX, MPH-ER) versus IR-stimulants for children with suboptimal response to IR-stimulant treatment [33, 37, 38], and for non-stimulants (GXR-ER) as adjunctive therapy to stimulant monotherapy for children with suboptimal response to stimulants [36, 39].

The evidence from studies investigating the cost-effectiveness of behavior management strategies for younger children with ASD, however, is mixed. Early intervention programs for children with suspected ASD were cost-effective, but communication-focused therapy for preschool children with ASD was not. The latter required a substantial investment of healthcare resources and did not improve health or result in cost savings in the healthcare or other sectors.

Although of moderate-to-good quality, the studies focusing on ADHD and ASD have important methodological differences, which reduce comparability. For instance, the analysis perspective determines the scope of the costs that are included in the analysis. Comparing the results of economic evaluations conducted from different perspectives can give different insights on how costs included impact on cost-effectiveness conclusions. Only four out of the ten studies targeting ADHD took a societal perspective and included costs outside the healthcare sector. This is a limitation of the current evidence base. Capturing the economic impacts of ADHD on relevant sectors of society is crucial for estimating the full economic value [46] of an intervention given the impact of ADHD on the educational sector, future earnings and employment of the child, and increased crime and substance abuse [6, 47, 48]. This is true for ASD as well, where school costs comprise the largest category for children, and productivity losses for parents and for children themselves as they become adults, are important costs related to the illness [8]. Taking narrower perspectives other than the societal may lead to recommendations that are detrimental to these children. All ASD studies in our review were conducted from the societal perspective.

In addition, no studies in our review captured the impact of ADHD or ASD on parents’ health and quality-of-life. Current economic evaluation guidelines from the USA [49], Canada [31], the UK [30], and the Netherlands [50] recommend the inclusion of family costs and health “spillover effects” when relevant. Both ASD and ADHD can substantially impact parents’ quality-of-life and mental health [51]. As a result, these impacts should be included in economic evaluations that focus on these disorders. Including family spillover effects in CEAs can meaningfully change the value of an intervention [52]. In a review of pediatric CEAs, the inclusion of family spillover effects in the evaluation made the cost-effectiveness of interventions more favorable 75% of the time [53].

The time horizon of the analyses was also quite heterogeneous, with most studies looking at costs and outcomes over short timeframes. Like study perspective, time horizon can have substantial influence on the results of an economic evaluation. On average, extending the time horizon of economic evaluations leads to more favorable estimates of value [54], and this is particularly important when the impact of an intervention may extend into the future, as is the case for most psychosocial/behavioral interventions for ASD and ADHD. Often, however, trials do not have follow-up periods that are long enough to evaluate how long the effectiveness of an intervention persists over time. There is also a paucity of data from other sources to be able to model the longer term costs and consequences of child/adolescent ADHD or ASD into adulthood. Capturing health and economic impacts over the long-term would provide better grounds to decision-making, considering the known impacts of ADHD and ASD across the individual’s life span.

In our review, three studies calculated QALYs using direct methods, and four used indirect methods. Cost-effectiveness guidelines in most countries recommend indirect methods, and the use of a generic HRQoL instrument to measure QALYs [30, 31]. In the context of ASD and ADHD, however, the most common instruments used to measure QALYs may not be appropriate. For example, the EQ5D measures HRQoL based on 5 domains of health: mobility, self-care, usual activities, pain/discomfort, and anxiety/depression [55]. This instrument may not fully capture the elements of HRQoL most relevant to children with ASD, including social, communication, and behavior problems, or ADHD, including inattention, hyperactivity, and impulsivity. Importantly, instruments such as the EQ5D have not been validated for use in children and adolescents. Although the EQ5D is recommended by international guidelines as the preferred method for measuring utilities in adults, no specific recommendations have been given on preferred instruments for measuring utilities in younger populations [30]. A few MAUIs exist that have been developed or adapted for use in younger populations. Examples are the HUI, the EQ5D-Y (youth version), the 16D and 17D, the Assessment of quality-of-life 6 Dimensions (AQoL-6D) Adolescent version and the Child Health Utility 9 Dimensions (CHU9D) [56]. Although many have been used in clinical and public health intervention studies to estimate QALYs for younger populations across different diseases [56], several methodological differences exist among them in terms of recommended age for application, dimensions included, and methods and populations used to derive utilities. For young populations with ASD/ADHD, appropriate MAUIs should include dimensions relevant to these populations, and although existing MAUIs, such as the 16D, 17D, AQoL-6D and CHU9D, cover a few aspects related to mental health, they may miss specific disease related changes. Importantly, no MAUI currently exists for assessing HRQoL in children younger than the age of five. Future research should focus on employing and developing instruments to capture meaningful changes in outcomes for the NDD population.

There is a need for the use of economic evaluations to assess the value of interventions for children with NDDs, a population with increasing demands for healthcare and other societal services [57]. This review revealed the limited information we currently have on the cost-effectiveness of interventions for ADHD and, in particular ASD. In addition, the limitations in methods used in the available studies are in line with a recent overview of economics and mental health by Knapp et al. [58], emphasizing similar shortcomings, including narrow costing perspectives, short follow-up periods, and lack of inclusion of “spillover effects” on carers and family. Given the health and economic burden of ASD and ADHD, more high-quality health economic data are needed to allow decision makers to develop policies and guidelines promoting cost-effective and clinically effective interventions for these children and their families.
